# Where Dopaminergic and Cholinergic Systems Interact: A Gateway for Tuning Neurodegenerative Disorders

**DOI:** 10.3389/fnbeh.2021.661973

**Published:** 2021-07-22

**Authors:** Marianne Amalric, Tommy Pattij, Ioannis Sotiropoulos, Joana M. Silva, Nuno Sousa, Samira Ztaou, Cristiano Chiamulera, Lars U. Wahlberg, Dwaine F. Emerich, Giovanna Paolone

**Affiliations:** ^1^Centre National de la Recherche Scientifique (CNRS), UMR 7291, Laboratoire de Neurosciences Cognitives, Aix-Marseille University (AMU), Marseille, France; ^2^Amsterdam Neuroscience, Department of Anatomy and Neurosciences, Amsterdam University Medical Centers, Amsterdam, Netherlands; ^3^Life and Health Sciences Research Institute (ICVS), School of Medicine, University of Minho, Braga, Portugal; ^4^ICVS/3B’s – PT Government Associate Laboratory, Braga, Portugal; ^5^Department of Molecular Therapeutics, New York State Psychiatric Institute, Department of Psychiatry, Columbia University, New York, NY, United States; ^6^Department of Diagnostic and Public Health, Section of Pharmacology, University of Verona, Verona, Italy; ^7^Gloriana Therapeutics, Inc., Warren, RI, United States; ^8^Independent Researcher, Glocester, RI, United States

**Keywords:** acetylcholine, dopamine, Alzheimer’s and Parkinson’s disease, impulse control, encapsulated cell-based system

## Abstract

Historically, many investigations into neurodegenerative diseases have focused on alterations in specific neuronal populations such as, for example, the loss of midbrain dopaminergic neurons in Parkinson’s disease (PD) and loss of cholinergic transmission in Alzheimer’s disease (AD). However, it has become increasingly clear that mammalian brain activities, from executive and motor functioning to memory and emotional responses, are strictly regulated by the integrity of multiple interdependent neuronal circuits. Among subcortical structures, the dopaminergic nigrostriatal and mesolimbic pathways as well as cholinergic innervation from basal forebrain and brainstem, play pivotal roles in orchestrating cognitive and non-cognitive symptoms in PD and AD. Understanding the functional interactions of these circuits and the consequent neurological changes that occur during degeneration provides new opportunities to understand the fundamental inter-workings of the human brain as well as develop new potential treatments for patients with dysfunctional neuronal circuits. Here, excerpted from a session of the European Behavioral Pharmacology Society meeting (Braga, Portugal, August 2019), we provide an update on our recent work in behavioral and cellular neuroscience that primarily focuses on interactions between cholinergic and dopaminergic systems in PD models, as well as stress in AD. These brief discussions include descriptions of (1) striatal cholinergic interneurons (CINs) and PD, (2) dopaminergic and cholinergic modulation of impulse control, and (3) the use of an implantable cell-based system for drug delivery directly the into brain and (4) the mechanisms through which day life stress, a risk factor for AD, damage protein and RNA homeostasis leading to AD neuronal malfunction.

## Introduction

Although research in neurodegenerative disorders have been focusing for many years on individual neuronal circuits and neurotransmitter systems [e.g., dopaminergic one in Parkinson’s disease (PD) and cholinergic in Alzheimer’s disease (AD)], it is increasingly accepted that different neurotransmitter systems are interrelated and affected under neurodegenerative conditions leading to deficits in related brain functions.

For instance, while PD research is commonly focused on the motor deficits resulting from the loss of nigrostriatal dopaminergic neurons (Paolone et al., 2015), a majority of PD patients suffer from non-motor symptoms such as cognitive and emotional disorders (Chaudhuri et al., 2006). These disturbances are, at least in part, related to a loss of basal forebrain cholinergic neurons but also increased cholinergic tone within the striatum, which temporally coincides with the loss of midbrain dopaminergic neurons (Bonsi et al., 2011; Yarnall et al., 2011). Many PD patients also have a tendency to fall and suffer from a freezing of gait, impairments in posture control and movement efficacy that are not treatable with L-DOPA. Relative to controls and non-falling patients, these individuals have greater reductions of cortical cholinergic activity (Bohnen et al., 2018). Preclinical studies support these findings with demonstrations that a concomitant loss of cholinergic and striatal dopamine afferents disrupts posture control and movement efficacy (Kucinski et al., 2013).

Similarly, memory impairment is the cardinal feature of AD, yet the clinical symptoms of this disorder also include a marked loss of motor function. Moreover, many AD and PD patients suffer from mood deficits, such as depression, a disease state where deficits in monoamines (e.g., dopamine), are found.

It is increasingly recognized that brain functions, from the executive and motor functioning to memory and emotional responses, are strictly regulated by the integrity of multiple interdependent neuronal circuits, the above clinical profiles in PD and AD, neurodegenerative disorders with different etiology, are likely the result of an intricate interplay of multi-system degenerations extending beyond the loss of nigrostriatal dopaminergic neurons in PD and the cholinergic denervation in AD (Paolone, 2020; Policastro et al., 2020).

To this aim, this mini-review will briefly discuss the main topics covered in a symposium from the 18th biennial meeting of the European Behavioral Pharmacology Society held in Braga, Portugal in August 2019 that focused on our current state of knowledge regarding functional interactions and cooperation of cholinergic and dopaminergic systems in motor and non-motor behaviors, as well as innovative developments in intracranial drug delivery in PD models and new insights into the role of chronic stress in AD models. This mini-review will start with describing electrophysiological and behavior data demonstrating the involvement of striatal cholinergic interneurons (CINs) in murine PD models, followed by a brief discussion on behavioral data indicating dopaminergic and cholinergic modulation of impulse control in rats. Subsequently, a novel encapsulated cell-based system for neurotrophic delivery directly into the brain will be introduced that demonstrated therapeutic effects in neurological and degenerative diseases. The mini-review will end with cellular data highlighting the effects of chronic stress on the dysregulation of proteostasis and RNA homeostasis in AD.

## Striatal Cholinergic Interneurons and Parkinson’s Disease

Marianne Amalric provided an update on the role of striatal CINs in the expression of motor, cognitive and motivational impairments in neurological disorders (Bonsi et al., 2011). The degeneration of nigrostriatal dopaminergic (DA) neurons in PD leads to an imbalance between the dopaminergic neuronal and CINs activity in the striatum that is thought to be associated with the emergence of rigidity, tremor and bradykinesia (Obeso et al., 2000; Aosaki et al., 2010). Anticholinergic drugs were the first therapeutic treatment for PD suggesting that an increased cholinergic tone in the striatum could result from striatal DA denervation (Duvoisin, 1967). Despite comprising less than 2% of all striatal neurons, they are potent modulators of medium spiny neuronal (MSNs) excitability, due to their widespread connections to output neurons. Modulation of MSNs by CINs may therefore appears as a critical player to reduce the imbalance between striatal DA and ACh activity (Calabresi et al., 2006; Pisani et al., 2007).

Although PD is traditionally classified as a movement disorder, it is increasingly recognized that non-motor symptoms frequently appear in the early stages or even during the pre-motor phase of the disease (Chaudhuri et al., 2006; Aarsland, 2016). A variety of non-motor symptoms, ranging from neuropsychiatric to cognitive impairments and loss of inhibitory control, are commonly observed in Parkinsonian patients. Although reciprocal interaction of acetylcholine and dopamine may underlie motor symptoms observed in pathophysiological conditions (Aosaki et al., 2010; Lester et al., 2010; Gittis and Kreitzer, 2012; Rizzi and Tan, 2017), much less is known on DA/Ach interactions in non-motor functions in the early stages of the disease. By combining a series of optogenetics, electrophysiological and pharmacological studies, Amalric and colleagues investigated the impact of striatal DA denervation in rodent models of PD on striatal CINs reactivity and behavioral outcome. The activity of striatal CINs is mainly driven by dopaminergic modulatory inputs and excitatory glutamatergic cortical and thalamic inputs. Nicotinic and muscarinic receptors are expressed at different levels of the striatal microcircuit where they modulate striatal afferent and efferent neuronal systems. In particular, the high level of expression of muscarinic acetylcholine receptors (mAChRs) in the striatum raised the question of their role in the regulation of the striatal network. *In vitro* studies in animal models of PD reveal that DA denervation of the striatum increases CINs excitability (Fino et al., 2007) and ACh release (Duvoisin, 1967; Bonsi et al., 2011) and contributes to the reorganization of striatal microcircuitry (Tozzi et al., 2016). The impact of CINs modulation *in vivo* on motor and non-motor symptoms in rodent models of PD is less known, however. Therefore, Amalric and colleagues investigated how optogenetic manipulation of CINs may affect the basal ganglia circuitry in different murine models of PD and how it translates to behavioral changes. To specifically express the opsins in striatal CINs, they performed stereotaxic injections of a Cre-inducible adeno-associated virus (AAV) vector carrying the gene encoding channelrhodopsin (ChR2) or halorhodopsin (eNpHR) into the striatum of transgenic mice expressing Cre-recombinase under the choline acetyltransferase (ChAt) promoter. *In vitro* recordings of CINs and MSNs revealed that photoactivation of ChR2 increased CINs firing activity in a light-locked manner while photoactivation of the inhibitory eNpHR opsin reduced firing activity. *In vivo* electrophysiological results in anesthetized mice, showed a normalization of the abnormal firing activity measured in the substantia nigra *pars reticulata*, the main output structure of the basal ganglia, in Parkinsonian conditions. Furthermore, it was found that photoinhibition of CINs activity primarily affected the transfer of cortico-striatal information by enhancing the activity of the direct striatonigral pathway, rather than reducing the activity of the indirect pathway (Maurice et al., 2015). Behavioral studies confirmed the critical contribution of striatal CINs in the various rodent models of late PD stage. In a pharmacological model (neuroleptic-induced catalepsy), photoinhibition of CINs reduced the akinetic symptoms, while their photoactivation did not modify the cataleptic behavior. In the lesional model of late PD (extensive DA lesions), CINs photoinhibition reversed all the asymmetric motor deficits, while the same optogenetics manipulation was ineffective in sham-control animals (Maurice et al., 2015; Ztaou et al., 2016). In a model of early PD stage, low dosage of the neurotoxin 6-OHDA induces an average of 30–40% loss of nigral DA neurons affect short-term memory in object and social recognition tests (Bonito-Oliva et al., 2014; Ztaou et al., 2016, 2018). Emotional deficits are also measured in the elevated cross maze in partially lesioned mice. CINs photoinhibition of transgenic mice expressing eNpHR in cholinergic neurons with similar partial 6-OHDA lesions alleviated the social recognition and cognitive deficits and reduced anxiety level, but did not affected the behavior of non-lesioned animals. These results suggest that even with a moderate striatal DA depletion, CINs reactivity may account for the cognitive and emotional symptoms measured in lesioned mice. Reducing their activity locally in the striatum may thus appear to be an alternative therapeutic target to reduce non-motor symptoms early in the disease in addition to alleviate motor impairments in the late-stage of PD (Ztaou et al., 2018; Ztaou and Amalric, 2019).

To decipher the mechanisms of ACh action on striatal postsynaptic M1 and M4 mAChRs, additional experiments were performed to pharmacologically block these receptors in normal and mutant mice lacking M4 receptors specifically in direct pathway MSN-D1 neurons (M4-D1 knockout mice). Blocking either M1 or M4 mAChRs in the dorsal striatum with telenzepine and tropicamide (M1 and M4 mAChR antagonists, respectively) reproduced the beneficial effect of optogenetics manipulation of CINs on motor symptoms. Interestingly, tropicamide had no effect in M4-D1 knockout mice. Postsynaptic M4 receptors expressed on direct MSNs output pathway may thus be preferentially involved in tropicamide action (Ztaou et al., 2016). The occurrence of motor and non-motor symptoms in PD may thus involve cholinergic activation of M1 and M4 muscarinic receptors of the striatum.

In summary, optogenetic inhibition of striatal CINs alleviates motor and non-motor deficits in rodent models of early and late PD stages. Optogenetic modulation of striatal CINs may thus provide new tools to treat both motor and cognitive symptoms of Parkinsonian patients.

## Dopaminergic and Cholinergic Modulation of Impulse Control

Tommy Pattij described their efforts to elucidate the roles of DA and ACh function in impulse control, noting that impulse control disturbances are important features in psychiatric disorders such as attention-deficit/hyperactivity disorder and substance use disorder (Moeller et al., 2001). In particular, he focused on inhibitory response control as one of the behavioral and cognitive phenomena of impulse control (Bari and Robbins, 2013). In view of this mini-review it is important to note that impulse control disturbances can also develop as non-motor symptoms in PD, and particularly can arise as a result of dopamine replacement therapy (Seppi et al., 2019). These impulse control disorders can develop in up to one out of five PD patients on dopamine replacement therapy and can manifest themselves as, for example, compulsive buying, hypersexual behavior and pathological gambling (Weintraub and Claassen, 2017).

There is an extensive preclinical literature on dopamine modulation of impulse control, that started decades ago with the observation that challenges with the psychostimulant amphetamine impair inhibitory response control (Cole and Robbins, 1987). Since then, many other studies have further elaborated on this and earlier work from Pattij and colleagues demonstrated the critical involvement of DA and, more specifically, of dopamine D1-like and dopamine D2-like receptors in inhibitory response control (Van Gaalen et al., 2006). Subsequent functional neuroanatomical approaches, including intracranial microinfusions of dopamine ligands and sophisticated rodent micro-positron emission tomography (PET) studies with dopamine D2/D3 ligands, have pinpointed the ventral striatum as a main brain region where dopamine D1-like and dopamine D2-like receptors modulate impulse control (e.g., Dalley et al., 2007; Pattij et al., 2007; Pezze et al., 2009; Besson et al., 2010; Caprioli et al., 2013; Jupp et al., 2013; Pattij and Vanderschuren, 2020). Importantly, the pre-clinical data are paralleled by clinical observations. Recent PET work found that human trait impulsivity correlates with enhanced amphetamine-evoked DA release in the ventral striatum and lower dopamine D2/D3 receptor availability in the midbrain (Buckholtz et al., 2010) and, moreover, with lower dopamine transporter availability in the ventral striatum (Smith et al., 2019). Thus, collective preclinical and clinical data have uncovered a striatal dopamine D2-like receptor mechanism subserving impulse control.

With regard to cholinergic modulation of impulsivity, pharmacological challenges with nicotine impair inhibitory response control (Hahn et al., 2002; Kolokotroni et al., 2011; Wiskerke et al., 2012), an effect that appears to depend on DA receptor activation (Van Gaalen et al., 2006). Thus, a functional interaction between the ACh and DA neurotransmitter system explains the effects of nicotine on impulse control. As such, it is well known that activation of somatodendritic nicotinic receptors on DA neurons in the ventral tegmental area evokes DA release in the ventral striatum explaining this functional interaction (e.g., Imperato et al., 1986; Barik and Wonnacott, 2009). Interestingly, although different from the acute pharmacological effects of nicotine on the brain, subchronic adolescent but not adult nicotine exposure resulted in long-lasting impairments in inhibitory response control as well as disturbances in attention in rats (Counotte et al., 2009, 2011). Strikingly, these adolescent nicotine effects on impulse control were accompanied by increased electrically-evoked DA release from the prefrontal cortex and not ventral striatum (Counotte et al., 2009). Further work pinpointed terminals of glutamatergic synapses in the medial prefrontal cortex as the brain locus where adolescent nicotine could have impacted impulse control and attention (Counotte et al., 2011). Recent optogenetic approaches have provided further insight into the roles of basal forebrain cholinergic neurons and prefrontal cortical CINs in inhibitory response control and attention. For this, the inhibitory opsin archaerhodopsin was expressed in ChAt-expressing interneurons in either the medial prefrontal cortex or basal forebrain cholinergic neurons in transgenic rats expressing Cre-recombinase under the ChAt promoter. As such this approach in rats is complementary to the murine PD-model experiments conducted by Amalric and colleagues described above (Maurice et al., 2015; Ztaou et al., 2016). Optical inhibition of basal forebrain cholinergic projections to the mPFC as well as CINs in the prefrontal cortex reduced attentional function, albeit at different time scales (Obermayer et al., 2019). In the same study, inhibitory response control was not affected by inhibiting the activity of either of these two types of ChAT containing neurons. Taken together, these results highlight the interplay between the dopaminergic and cholinergic neurotransmitter systems in modulating impulse control, either by activation of nicotinic ACh receptors on DA neurons or on terminals of glutamatergic synapses.

## Encapsulated Cell Therapy: Targeting Dopaminergic and Cholinergic Structural Alterations With Neurotrophic Factors as a New Strategy in the Pathophysiology of Neurodegenerative Disorders

Giovanna Paolone has exploited an encapsulated cell technology that, following to implantation into the brain, provides a targeted, continuous, *de novo* synthesized source of proteins that can be distributed directly to the desired brain region (Lindvall and Wahlberg, 2008; Emerich et al., 2019; Paolone et al., 2019). These studies were based on the use of human ARPE-19 cells that had been genetically modified to produce trophic molecules including glial cell line-derived neurotrophic factor (GDNF), brain-derived neurotrophic factor (BDNF), and nerve growth factor (NGF). Prior to implantation, the cells were “encapsulated” within semipermeable, immunoisolatory hollow fiber membranes to facilitate their implantation, allow their retrieval for confirmation of function, and minimize immunological rejection. Initial studies evaluated the potential of GDNF in pre-clinical models of epilepsy. GDNF is a particularly interesting candidate for epilepsy as it is physiologically found within the temporal lobe, is upregulated in response to seizure activity, and local delivery can reduce seizures in animal models (Kanter-Schlifke et al., 2007). Extensive *in vivo* studies were conducted in a pilocarpine rat model of epilepsy. Animals with established seizures received bilateral implants of GDNF-secreting devices into the hippocampus and were tested on a battery of neurological tests over several months. Results included:

(1) Controlled, stable, and long-term (at least 6 months) delivery of GDNF to the hippocampus in a well-tolerated manner.
(2) GDNF significantly reduced (>90%) pilocarpine-induced seizures while also normalizing changes in anxiety-like and cognition over several months. In addition to reducing behavioral seizures, it was also found that GDNF significantly reduced seizures as measured by EEG.
(3) The benefits of GDNF were both symptomatic and disease-modifying as the reductions in seizures persisted even when the devices were retrieved.
(4) The functional benefits were associated with protection of the hippocampus against the pathological changes brain anatomy that accompany epilepsy, including hippocampal atrophy, cell degeneration, loss of parvalbumin-positive interneurons, and abnormal neurogenesis. The neuronal protection was associated with GDNF receptor activation (Paolone et al., 2019).

The versatility of this system was confirmed when similar benefits were observed when delivering BDNF to the temporal lobe of pilocarpine-treated rats. In these studies, the frequency of spontaneous seizures was reduced by more than 80%, cognitive performance was improved, and the neurological benefits of BDNF were associated with reductions in degenerating cells and normalization of hippocampal volume and neurogenesis (Falcicchia et al., 2018).

Dr. Paolone further described studies using GDNF as a potential treatment for PD. While GDNF has a relatively long and promising pre-clinical history as a potent neuroprotective agent in models of PD (Choi-Lundberg et al., 1997; Kordower et al., 2000; Kirkeby and Barker, 2019; Whone et al., 2019) its clinical utility has been difficult to test. To be effective, GDNF needs to be delivered selectively in a long-term and stable manner while covering the nigrostriatal system. Implants of encapsulated GDNF cells one week prior to intrastriatal 6-OHDA injections in rats protected DA neurons in the substantia nigra, preserved DA fibers in the striatum and protected against declines in motor performance. To quantify behavioral extent of the lesion as well as the benefits of GDNF implants, rats behavior was assessed prior to device implant, prior to 6-OHDA lesion and again two and four weeks post lesion using the cylinder, placing and stepping test. When cell-based delivery of GDNF occurred four weeks post 6-OHDA lesions (i.e., a neurorestorative model), improvement in the forelimb use was observed as early as four weeks post GDNF treatment and continued to grow for over one year (62 weeks). Similarly, impressive distribution of GDNF and positive effects on DA function were observed when larger, clinical-sized devices were implanted for three months into the putamen of minipigs. Implantation of two devices, separated by 5 mm, resulted in distribution of GDNF throughout the putamen and caudate that robustly upregulated the expression of tyrosine hydroxylase staining in the regions covered by GDNF diffusion (Wahlberg et al., 2020).

Although the mechanisms are not completely understood, proper function of cholinergic neurons located in the basal forebrain, relies on the supply of NGF retrogradely transported from the cortex and hippocampus (Salehi et al., 2004). Neurons in the medial septal nucleus, the nucleus of the diagonal band of Broca, the nucleus basalis of Meynert, and the substantia innominata, including their cortical and hippocampal projections are severely lost in AD contributing to memory and attention deficits. In rats, NGF cells survive long-term (1 year) and protect cholinergic cells in lesioned and aged animals (Winn et al., 1994). Similarly, in non-human primate, NGF protects septal neurons in lesioned and aged monkey (Emerich et al., 1994; Kordower et al., 1994, 1996). The safety and tolerability of this technology as well as the biological effects, have also been explored in patients with mild to moderate AD to deliver NGF directly to the basal forebrain to restore cholinergic function (Wahlberg et al., 2012; Ferreira et al., 2015; Karami et al., 2015).

In rats, performance of a Sustained Attention Task (SAT) induces a performance-associated increase in cortical cholinergic neurotransmission depending on the integrity of the cholinergic inputs to the prefrontal or posterior parietal cortex. Furthermore, attentional performance is enhanced by the stimulation of the mesolimbic circuitry, particularly the shell of the nucleus accumbens (NAc) through the activation of basal forebrain corticoperal projections (St Peters et al., 2011; Paolone et al., 2012, 2013).

Given that these results support the potential use of encapsulated trophic factor-secreting cells in human diseases such as PD, AD and epilepsy, future studies might focus on the simultaneous delivery of multiple factors to more fully treat the pathology mosaicism that occurs in multisystem disorders such as neurological diseases.

## The Interplay of Cholinergic Innervation and Chronic Stress in AD Neuropathology

Investigation in PD provided a model for the pursuit of the selective neuronal vulnerability in the AD brain which was originally focused on cholinergic neurons. In 1970’s, the first evidence suggested a selective reduction of the activity of the acetylcholine synthetic enzyme choline acetyltransferase (ChAT) as well as the acetylcholinesterase (AChE) in the brain area of hippocampus, a region known to participate in memory functions, as well as in cortex and amygdala (Davies and Maloney, 1976) while other studies described a relationship between ChAT activity and mental ability in demented subjects (Perry et al., 1978). Today, it is widely accepted that cortical cholinergic denervation in the AD brain represents one of the earliest and most severe transmitter changes while drugs that boosting cholinergic system (e.g., by AChE inhibition) are widely used for mild/moderate AD patients. Overall, the cholinergic hypothesis has been implicated in the AD etiology and it is based on the degeneration of cholinergic neurons of basal forebrain which can cause memory deficits. Interestingly, the cholinergic system is also involved to the response to stress and the regulation to stress-related hypothalamic-pituitary-adrenal (HPA) axis (Saswati et al., 2015) while loss of cholinergic input to the hippocampus is suggested to induce AD hippocampal vulnerability aggravating memory deficits caused by stress (Craig et al., 2011). Thus, the work described by Dr. Ioannis Sotiropoulos in the EBPS 2019 meeting focused on the recent evidence about the interplay of chronic stress and AD on novel neurodegenerative mechanisms in hippocampus with particular attention on Tau protein which seems to be the converging protein between chronic stress and AD brain pathologies. For instance, exposure to chronic stress or high levels of major stress hormones including glucocorticoids (GC) increases the levels of aberrantly hyperphosphorylated Tau together with neuronal atrophy, synaptic malfunction, reduced neurogenesis, and memory deficits (Sotiropoulos et al., 2011; Lopes et al., 2016; Dioli et al., 2017; Pedrazzoli et al., 2019). Importantly, the hyperphosphorylation occurred at certain Tau epitopes that are strongly implicated in cytoskeletal dysfunction and synaptic loss (e.g., pSer262) (Callahan et al., 2002) and hippocampal atrophy (e.g., pThr231) (Hampel et al., 2005) in AD patients. Related to synaptic malfunction and loss, chronic stress causes the missorting of hyperphosphorylated Tau to synapses which subsequently become dysfunctional (Lopes et al., 2016; Pinheiro et al., 2016). The missorting of Tau to synapses is now acknowledged as an early event in AD, preceding the manifestation of detectable neurodegenerative processes related to excitotoxic synaptic signaling and malfunction (Ittner et al., 2010). Intriguingly, Tau deletion prevents the aforementioned stress-induced signaling as well as neurostructural and behavioral deficits (Lopes et al., 2016), suggesting that Tau is the “final executor” of stress/GC induced neurotoxicity, similar to the reported role for Tau as a mediator of Aβ-driven neurotoxicity in AD (Ittner et al., 2010).

*In vitro* and *in vivo* studies suggest that stress and GC reduce the degradation of Tau in hippocampus, thereby increasing its accumulation (Sotiropoulos et al., 2008) *via* dysregulation of molecular chaperones (responsible for Tau proteostasis) (Sotiropoulos et al., 2015). More recent efforts have focused on the impact of chronic stress and high GC on two essential degradative mechanisms of Tau, the endolysosomal pathway (Vaz-Silva et al., 2018) and autophagy (Silva et al., 2018). The endolysosomal pathway has been implicated in neurodegenerative diseases such as AD and PD in which Tau accumulation is a pathological feature (Kett and Dauer, 2016; Small et al., 2017). Current work by Sotiropoulos and colleagues has identified Tau as a substrate of the endolysosomal degradation pathway (Vaz-Silva et al., 2018) while it demonstrated that *in vitro* or *in vivo* exposure to high GC levels blocks this pathway, accompanied by the accumulation of Tau. Further, they showed that the involvement of the small GTPase, Rab35, and the endosomal sorting complexes required for transport (ESCRT) machinery that delivers Tau to lysosomes *via* early endosomes and multivesicular bodies (MVBs). Importantly, high GC suppress *Rab35* transcription resulting in Tau accumulation due to its impaired degradation while overexpression of Rab35 reverses GC-induced Tau accumulation and related neuronal atrophy in the hippocampus (Vaz-Silva et al., 2018). Based on the suggested signaling interplay between cholinergic and GC receptors, future studies should monitor whether cholinergic signaling participates in this GC action on the endolysosomal degradation pathway.

Though its ability to degrade long-lived and misfolded proteins such as Tau, autophagy and its interruption is causally related to the accumulation of Tau protein aggregates in the AD brain. Recent studies presented by Dr. Sotiropoulos at EBPS meeting demonstrated for the first time, that both, chronic stress and high GC levels inhibit the autophagic process *via* activation of mTOR signaling providing another mechanism through which these conditions contribute to the accumulation and aggregation of Tau and downstream neurodegeneration (Silva et al., 2018). These findings are in line with previous reports that chronic stress stimulates mTOR activity (Polman et al., 2012), an event associated with increased total Tau levels in the brains of AD subjects (Pei and Hugon, 2008). Furthermore, inhibition of mTOR signaling is shown to ameliorate Tau pathology (Jiang et al., 2014) while our studies show that inhibition of mTOR blocked the GC-driven Tau accumulation and aggregation (Silva et al., 2018). Interestingly, autophagy is related to the degradation of stress granules (SG) that are conserved cytoplasmic aggregates of ribonucleoprotein complexes (RNPs) implicated in the regulation of RNA translation, storage, and decay (Wolozin and Ivanov, 2019). While the formation of SGs is considered a protective mechanism against cellular stress (e.g., oxidative stress), prolonged SG induction can become pathological and neurotoxic. For instance, in AD neurodegeneration, SG promote the accumulation of Tau aggregates in a vicious cycle wherein Tau stimulates SG formation, with the RNA binding protein TIA1 playing a lead role in Tau misfolding and aggregation (Wolozin and Ivanov, 2019). Dr. Sotiropoulos showed that chronic stress and high GC increase the protein levels of various RBP and SG markers in soluble and insoluble fractions in both cellular and animal models of Tau pathology. Specifically, chronic stress increased cytoplasmic (soluble and insoluble) levels of several RBPs and SG-associated markers (e.g., TIA-1, PABP, G3BP, FUS, DDX5) that contributed to the formation of insoluble Tau inclusions and Tau accumulation (Small et al., 2017). As noted above, TIA-1 plays a prominent role in Tau aggregation (Vanderweyde et al., 2016; Apicco et al., 2018). Under stressful conditions, TIA-1 is trafficked from the nucleus to the cytospasm where it interacts directly with Tau (and other RBPs) to stimulate its aggregation and accumulation (Pei and Hugon, 2008). Tau missorting and accumulation in the dendritic compartment, such as is found in AD pathology, is also triggered by chronic stress/GC exposure (Lopes et al., 2016; Pinheiro et al., 2016). Thus, the above findings highlight the important role of chronic stress and GC signaling in the hippocampal neurodegeneration in AD brain adding to the suggested complexity between different factors/parameters that contribute to precipitates of AD brain pathology.

## Concluding Remarks

This mini-review briefly describes recent developments in behavioral and cellular neuroscience as part of a symposium outcome and indicates that neurodegenerative diseases such as PD and AD have complex, multi-system changes in neuronal circuits that underlie the disease’s characteristic neurobehavioral changes. Our understanding of the molecular, neurochemical, intraneuronal, and circuitry pathology underlying these diseases has advanced considerably with developments in analytical techniques and convergences in disciplines including model development, molecular biology, engineering, and pharmacology. Highlighted in this mini-review is the importance of continued refinements in behavioral pharmacology where understanding the functional consequences of disease manifestation will lead to more rapid developments in medical advancements. In particular, the presented optogenetic data from transgenic mice and rats expressing Cre-recombinase under the ChAt promoter indicate (1) the interplay between the CINs and dopaminergic system in the striatum in motor and non-motor behavior in murine 6-OHDA-PD models, as well as (2) new insights into cholinergic modulation of attention in the prefrontal cortex by directly comparing basal forebrain cholinergic inputs and CINs in this cognitive function. Novel intracranial drug delivery methods have revealed neuroprotective effects of GDNF and NGF on dopamine and achetylcholine degeneration.

The novel and powerful tools of regulation of cholinergic and dopaminergic innervation would offer novel and solid evidence about their individual contribution in neuronal pathology and behavioral impairment in different brain areas and circuits of the neurodegenerative brain in different stages of the disease.

## Author Contributions

MA, TP, IS, DE, and GP wrote the manuscript. MA, TP, IS, JS, NS, SZ, DE, and GP were involved in the conceptualization of the studies. All authors contributed to the article and approved the submitted version.

## Conflict of Interest

LW is the CEO of Gloriana Therapeutics, Inc., a for-profit biotechnology company that is developing the encapsulated cell technology to treat CNS diseases. The remaining authors declare that the research was conducted in the absence of any commercial or financial relationships that could be construed as a potential conflict of interest.
